# Timing and duration of diabetes and incident dementia in older adults: a prospective cohort study

**DOI:** 10.1186/s13195-026-02189-z

**Published:** 2026-10-10

**Authors:** Lingyan Zhao, Hanbo Yu, Ruihao Song, Lin Wang, Youlie Yang, Xiaomei Wang, Jing Fan, Guiping Li

**Affiliations:** 1 First Teaching Hospital of Tianjin University of Traditional Chinese Medicine, No.88 Changling road, Xiqing District, Tianjin city, 300381 China; 2 National Clinical Research Center for Chinese Medicine, Tianjin city, 300381 China; 3 Qingdao Central Hospital, University of Health and Rehabilitation Sciences, Qingdao city, 266042 China

**Keywords:** Diabetes, Dementia, Life-course epidemiology, Landmark analysis

## Abstract

**Background:**

Diabetes is associated with dementia, but how this association varies with attained age and cumulative exposure remains unclear. We integrated time-updated exposure histories with repeated age-specific landmark cohorts, enabling complementary evaluation of overall and age-conditional dementia risk.

**Methods:**

We analysed 18,028 dementia-free participants aged 50 years or older from the Health and Retirement Study. Incident dementia was classified using the Langa–Weir algorithm. Time-updated Cox models evaluated risk according to current diabetes status. Age-anchored landmark models used delayed entry at six prespecified ages from 50 to 75 years. Formal age interactions and competing mortality were also assessed.

**Results:**

Diabetes was associated with higher dementia risk in the time-updated analysis (HR, 1.29; 95% CI, 1.19–1.41). The association attenuated with older attained age but remained evident across the landmark cohorts. An estimated interval exceeding 10 years since first diabetes ascertainment identified groups at higher risk. The findings remained consistent after accounting for competing death.

**Conclusions:**

Diabetes was associated with dementia throughout later life, with stronger associations at younger attained ages and among participants with a longer estimated interval since first ascertainment. These observational findings support considering the timing of diabetes ascertainment in cognitive risk assessment.

**Supplementary Information:**

The online version contains supplementary material available at https://doi.org/10.1186/s13195-026-02189-z.

## Introduction

Dementia is a major public health challenge in ageing populations. The Global Burden of Disease Study 2021 estimated that 56.9 million people were living with Alzheimer’s disease and other dementias in 2021, with 36.3 million disability-adjusted life-years and 1.95 million deaths attributable to these conditions [1]. Because disease-modifying therapies remain limited, identifying modifiable risk factors and the life stages in which they matter most is central to dementia prevention [2–5].

Diabetes is a well-established modifiable risk factor for cognitive impairment and dementia [6, 7]. Its association with dementia is unlikely to depend only on diabetes status at one assessment. Chronic hyperglycaemia may promote advanced glycation end products, oxidative stress, inflammation, and vascular endothelial dysfunction [8–10], which can impair the neurovascular unit and contribute to cognitive decline [11]. Diabetes-related dementia risk may therefore reflect age at onset and cumulative metabolic exposure, rather than current diabetes status alone.

Prior evidence supports the importance of timing and duration. In the Whitehall II cohort, younger age at diabetes onset and duration longer than 10 years were associated with higher subsequent dementia risk [12]. Other studies have reported similar associations for early-onset diabetes or longer type 2 diabetes duration [13, 14]. However, many studies have relied on baseline exposure status, broad onset-age categories, or selected populations. Fewer have characterized age-varying diabetes-dementia associations across multiple prespecified landmark ages in a nationally representative older population.

Among older adults, diabetes–dementia associations may also be shaped by death as a competing event and by heart disease or stroke as potential vascular intermediates. Diabetes is associated with heart disease, stroke and mortality [15, 16]. Competing death may alter estimates of dementia incidence, while cardiovascular or cerebrovascular disease may represent an intermediate association between diabetes and dementia.

Using the Health and Retirement Study (HRS), a nationally representative longitudinal cohort in the United States, we evaluated associations of diabetes status, assigned exposure-transition age and estimated time since first diabetes ascertainment with incident dementia. The time-updated Cox model addressed risk at participants’ current exposure values across follow-up. Complementary age-anchored landmark models estimated subsequent risk using delayed entry at prespecified ages. We also tested age modification formally, accounted for competing death and estimated exploratory indirect associations through heart disease or stroke.

## Methods

### Study design and data source

This prospective cohort study used six biennial Health and Retirement Study waves, waves 10 through 15, conducted from 2010 to 2020. The final analytical cohort included 18,028 participants at wave 10. Among these participants, 17,623, 16,082, 14,210, 11,678 and 10,687 contributed interview data at waves 11–15, respectively. Participation in all six waves was not required. Final analytic-cohort participation and wave-specific data availability are reported in Supplementary eTable 1.

Self-reported physician diagnosis of diabetes and glucose-lowering medication use were available at every attended interview. Dried-blood-spot HbA1c was available only in 2010, 2012, 2014, and 2016, corresponding to waves 10–13. HbA1c was not collected at waves 14 and 15.

### Participants and missing data

**Fig. 1:**
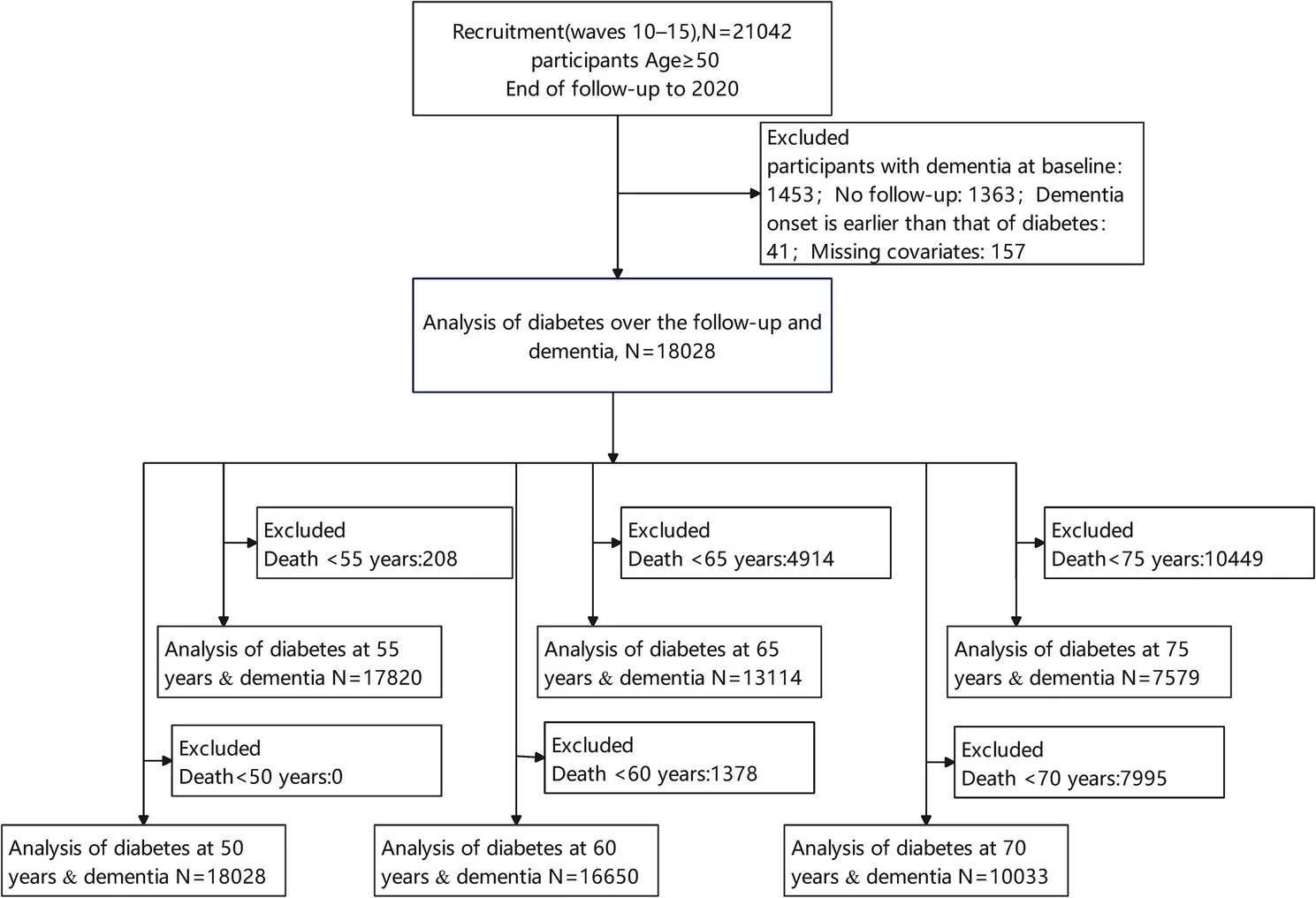
Participant selection for the primary and landmark analyses

Participants were eligible if they had wave 10 information, were aged 50 years or older, were free of dementia at baseline, and had follow-up information sufficient to determine incident dementia, death, or censoring. We excluded participants with baseline dementia, no valid post-baseline follow-up, missing key covariates, or dementia first ascertained before a later diabetes record. The final analytic cohort comprised 18,028 participants. The selection process is shown in Fig. 1.

### Cohort construction and missing data

The primary cohort excluded participants whose dementia was first ascertained before a later diabetes record. This restriction preserved the observed temporal ordering between diabetes ascertainment and incident dementia among participants classified as having diabetes. Complete-case analysis was used for the baseline covariates included in the multivariable models. Missing HbA1c alone did not result in exclusion and was not interpreted as absence of diabetes.

When a follow-up interview was missed or did not provide sufficient diabetes or cognitive information, no new exposure or outcome status was assigned at that wave. The next attended wave provided the subsequent assessment. Diabetes or dementia newly identified at that assessment was dated to the later assessment and was not retrospectively assigned to the missed wave. Once diabetes was ascertained, participants remained classified as exposed thereafter.

The HRS was approved by the Institutional Review Board of the University of Michigan, and participants provided written informed consent. This analysis used publicly available deidentified data and followed the STROBE reporting guideline.

### Diabetes definition and exposure timing

Diabetes status was assessed at each attended wave using self-reported physician-diagnosed diabetes, insulin or oral glucose-lowering medication use, or glycated haemoglobin (HbA1c) of 6.5% or higher when biomarker data were available. HbA1c data were available in waves 10–13 but were not collected in waves 14 or 15. In waves 10 and 11, HbA1c was measured by Heritage Laboratory using the Appraise^®^ dried-blood-spot HbA1c assay. In waves 12 and 13, HbA1c was measured at the University of Washington using the Bio-Rad Variant II high-performance liquid chromatography system. Thus, the laboratory and assay protocol differed between waves 10–11 and waves 12–13. To improve comparability across waves, the HbA1c threshold was applied to the HRS-provided NHANES-equivalent values rather than the unstandardised raw assay measurements. These harmonised values were used in all analyses involving HbA1c.

The dataset did not contain the exact date or age of clinical diabetes onset. We therefore used the date of the first attended interview or biomarker assessment meeting the diabetes criteria as a proxy diabetes ascertainment date. The assigned exposure-transition age was calculated from the year and month of that assessment and the participant’s year and month of birth. If an intermediate wave was missed or lacked sufficient diabetes information before first ascertainment, no exposure transition was assigned at that wave. A subsequent transition was assigned at the first later assessment meeting the diabetes criteria. Diabetes was not retrospectively assigned to the missed wave. Once diabetes was identified, participants remained classified as exposed thereafter.

Missing HbA1c was not interpreted as absence of diabetes. At waves without available HbA1c data, classification was based on self-reported physician diagnosis, glucose-lowering medication use and previously ascertained diabetes status. In the time-updated Cox model, participants contributed non-diabetes person-time until their assigned exposure-transition age and diabetes-exposed person-time thereafter.

For each landmark analysis, diabetes status was defined according to whether the assigned exposure-transition age occurred on or before the prespecified landmark age. Estimated time since first diabetes ascertainment was calculated as the landmark age minus the assigned exposure-transition age. It was categorised as 5 years or less, more than 5 to 10 years, or more than 10 years. Diabetes status and estimated time since first ascertainment were defined at the landmark age and held fixed during post-entry follow-up.

### Incident dementia

Dementia was classified at each attended wave using the Langa–Weir algorithm. Among self-respondents, dementia was defined as a score of 6 or lower on the 27-point HRS cognitive scale. Among proxy respondents, dementia was defined as a proxy cognitive score greater than 5. Cognitive assessment formed part of the standard biennial HRS interview and was not triggered by diabetes diagnosis or frequency of healthcare contact. Incident dementia was assigned to the first observed follow-up wave meeting the algorithmic criteria. This time therefore represents the first observed algorithm-positive assessment rather than an adjudicated clinical onset date. A missing cognitive classification was not treated as dementia. Participants could contribute cognitive information again after returning at a later wave. Follow-up ended at incident dementia, death, the last wave with ascertainable outcome status or wave 15.

### Covariates

Models included age, sex, race or ethnicity, education, marital status, smoking, alcohol consumption, cancer, and chronic lung disease. Cancer and chronic lung disease were selected as major noncardiovascular conditions reflecting general comorbidity and competing health burden.

Heart disease and stroke were excluded from the principal fully adjusted model because they could lie on a pathway linking diabetes with dementia. They were evaluated separately in supplementary and exploratory mediation analyses.

### Statistical analysis

Baseline characteristics were summarised according to incident dementia status. Continuous variables are presented as medians with interquartile ranges, and categorical variables as numbers and percentages. Between-group comparisons used the Wilcoxon rank-sum test or Pearson’s χ² test, as appropriate.

Cox proportional hazards models used attained age as the underlying time scale. Participants entered the risk set at their age at the wave 10 interview, thereby accounting for delayed study entry. Diabetes was modelled as a time-updated exposure using counting-process start–stop intervals. Participants with diabetes at wave 10 were classified as exposed from study entry. Participants first identified as having diabetes during follow-up were classified as not yet having ascertained diabetes before their first qualifying assessment and as having diabetes from that assessment onwards. The exposure-transition age was defined as age at the first attended interview or biomarker assessment meeting the diabetes criteria, as described above.

Separate age-anchored landmark Cox models were fitted at ages 50, 55, 60, 65, 70 and 75 years. Participants entered each risk set at the later of the prespecified landmark age or their age at the wave 10 interview. Those who had developed dementia or died before risk-set entry were excluded. Diabetes status was defined by whether the assigned exposure-transition age occurred on or before the prespecified landmark age. Estimated time since first diabetes ascertainment was calculated as the landmark age minus the assigned exposure-transition age and categorised as 5 years or less, more than 5 to 10 years, or more than 10 years. Both variables were held fixed during post-entry follow-up. Thus, the time-updated model estimated associations according to current diabetes exposure, while the landmark models estimated conditional associations within age-anchored, delayed-entry risk sets.

The proportional hazards assumption was assessed using scaled Schoenfeld residuals for the diabetes coefficient and the overall model. Effect modification by attained age was formally evaluated in the time-updated model using a diabetes-by-age interaction, expressed per 5-year increase in age. Differences across landmark ages were assessed using a pooled landmark model containing a diabetes-by-landmark-age interaction. The pooled model was stratified by landmark age and used participant-clustered robust standard errors to account for participants contributing to multiple landmark cohorts. Where non-proportionality was detected, the constant diabetes hazard ratio was interpreted as a follow-up-averaged association, with greater emphasis placed on the age-interaction estimate.

Models were adjusted sequentially. Model 1 included education, race or ethnicity, marital status and sex. Model 2 additionally included alcohol consumption and smoking status. Model 3 additionally included cancer and chronic lung disease as major non-vascular comorbidities that could be associated with overall health, mortality and dementia risk. Fine–Gray subdistribution hazards models treated death without dementia as a competing event.

Counterfactual mediation analyses evaluated a composite indicator of heart disease or stroke as a potential vascular intermediate. The models allowed an exposure–mediator interaction and estimated total, direct and indirect associations and the proportion mediated. Interpretation requires consistency and positivity, correct model specification and temporal ordering, no unmeasured exposure–outcome, exposure–mediator or mediator–outcome confounding, and no mediator–outcome confounder affected by diabetes. These assumptions cannot be fully verified in the observational, wave-based HRS data. The analyses were therefore considered exploratory and were not interpreted as establishing causal mechanisms.

Sensitivity analyses excluded participants who developed dementia within two years after risk-set entry and those whose diabetes classification was based solely on elevated HbA1c. Participants with baseline HbA1c measurements were also compared descriptively with the full analytic cohort to assess potential biomarker-subset selection.

Missing baseline covariates were handled using complete-case analysis. A missing HbA1c measurement was not interpreted as absence of diabetes. When an intermediate diabetes assessment was unavailable, diabetes status was not retrospectively assigned to the missed wave. The first subsequent qualifying assessment was instead used as the exposure-transition date. Cognitive classifications were obtained from the HRS Langa–Weir final imputed classification data, and no additional cognitive-data imputation was conducted. All tests were two-sided, and *P* < .05 was considered statistically significant. Analyses were performed using R.

## Results

### Participant characteristics

Among 18,028 participants free of dementia at baseline, 2247 developed dementia during follow-up. Participants who developed dementia were older, had shorter follow-up, and had less favourable socioeconomic and health profiles than those who did not develop dementia. The dementia group also had higher prevalences of diabetes, hypertension, heart disease, and stroke (Table 1). Characteristics of the six prespecified landmark cohorts, including participant counts, dementia events and estimated intervals since first diabetes ascertainment, are presented in eTable 2.

**Table 1:** Characteristics of participants according to dementia status at the end of follow-up

Characteristic	No dementia1	Dementia1	*p*-value2
Participants, *n*	15,781	2,247	
Age, years	62.0 (56.0, 72.0)	74.0 (64.0, 81.0)	< 0.001
Gender			0.003
Men	6,786 (43%)	891 (40%)	
Women	8,995 (57%)	1,356 (60%)	
Education			< 0.001
≤Partial secondary school	2,218 (14%)	865 (38%)	
High school diploma	5,398 (34%)	736 (33%)	
≥University degree	8,165 (52%)	646 (29%)	
Race			< 0.001
white/caucasian	11,737 (74%)	1,486 (66%)	
black/african american	2,784 (18%)	570 (25%)	
other	1,260 (8.0%)	191 (8.5%)	
Marital status			< 0.001
Married	9,689 (61%)	1,059 (47%)	
Other	6,092 (39%)	1,188 (53%)	
Smoker			0.101
never smoker	6,903 (44%)	950 (42%)	
Former smoker	6,443 (41%)	970 (43%)	
Current smoker	2,435 (15%)	327 (15%)	
Alcohol consumption			< 0.001
No	6,201 (39%)	1,326 (59%)	
Yes	9,580 (61%)	921 (41%)	
Health conditions			
Heart disease			< 0.001
No	10,362 (66%)	1,199 (53%)	
Yes	5,419 (34%)	1,048 (47%)	
Diabetes			< 0.001
No	10,241 (65%)	1,341 (60%)	
Yes	5,540 (35%)	906 (40%)	
Hypertension			< 0.001
No	3,641 (23%)	290 (13%)	
Yes	12,140 (77%)	1,957 (87%)	
Stroke			< 0.001
No	14,156 (90%)	1,638 (73%)	
Yes	1,625 (10%)	609 (27%)	
Chronic lung disease			< 0.001
No	13,307 (84%)	1,783 (79%)	
Yes	2,474 (16%)	464 (21%)	
Cancer			< 0.001
No	12,555 (80%)	1,696 (75%)	
Yes	3,226 (20%)	551 (25%)	
Death	3,972 (25%)	1,307 (58%)	< 0.001
Follow-up time, y	10.0 (7.0, 10.0)	4.0 (2.0, 8.0)	< 0.001

Values are median (IQR) for continuous variables and No. (%) for categorical variables. Dementia was defined using the Langa-Weir classification algorithm. *P* values were calculated using the Wilcoxon rank-sum test for continuous variables and Pearson chi-square test for categorical variables

*Abbreviation*: *IQR* Interquartile range

### Overall association between diabetes and incident dementia

In Cox models treating diabetes as a time-updated exposure, diabetes was associated with higher incident dementia risk (Table 2). The fully adjusted model yielded an HR of 1.29 (95% CI, 1.19–1.41; *P* < .001) after adjustment for sociodemographic characteristics, health behaviours, cancer and chronic lung disease.

**Table 2:** Association between time-updated diabetes status and incident dementia

Model	Follow-up, mean (SD), y	HR (95% CI)	*P* value	Participants / dementia events, No.
Model 1	8.04 (2.70)	1.32 (1.22–1.44)	< 0.001	18,028 / 2,247
Model 2	8.04 (2.70)	1.30 (1.19–1.42)	< 0.001	18,028 / 2,247
Model 3	8.04 (2.70)	1.29 (1.19–1.41)	< 0.001	18,028 / 2,247

Cox proportional hazards models used age as the underlying time scale with start–stop intervals. Diabetes status was modelled as a time-updated exposure

*HR* Hazard ratio, *CI* Confidence interval

### Diabetes status at prespecified landmark ages

Diabetes was associated with higher incident dementia risk at all prespecified landmark ages (Table 3). In fully adjusted models, HRs were 1.49 (95% CI, 1.26–1.75) at age 50 years, 1.53 (95% CI, 1.34–1.75) at age 55 years, 1.46 (95% CI, 1.28–1.66) at age 60 years, 1.31 (95% CI, 1.15–1.49) at age 65 years, 1.30 (95% CI, 1.15–1.48) at age 70 years and 1.28 (95% CI, 1.13–1.46) at age 75 years.

**Table 3:** Association of diabetes with incident dementia at prespecified landmark ages

Landmark age, y	Follow-up, mean (SD), y	Model 1, HR (95% CI)	*P* value	Model 2, HR (95% CI)	*P* value	Model 3, HR (95% CI)	*P* value	Participants / dementia events, No.
50	8.04 (2.70)	1.52 (1.29–1.79)	< 0.001	1.49 (1.26–1.76)	< 0.001	1.49 (1.26–1.75)	< 0.001	18,028 / 2,247
55	7.65 (2.73)	1.57 (1.38–1.80)	< 0.001	1.54 (1.34–1.76)	< 0.001	1.53 (1.34–1.75)	< 0.001	17,820 / 2,209
60	6.74 (3.07)	1.51 (1.32–1.71)	< 0.001	1.46 (1.28–1.66)	< 0.001	1.46 (1.28–1.66)	< 0.001	16,650 / 2,044
65	6.42 (3.12)	1.36 (1.19–1.55)	< 0.001	1.31 (1.15–1.49)	< 0.001	1.31 (1.15–1.49)	< 0.001	13,114 / 1,852
70	6.27 (3.09)	1.36 (1.20–1.54)	< 0.001	1.30 (1.15–1.48)	< 0.001	1.30 (1.15–1.48)	< 0.001	10,033 / 1,673
75	5.63 (2.90)	1.33 (1.17–1.51)	< 0.001	1.28 (1.13–1.46)	< 0.001	1.28 (1.13–1.46)	< 0.001	7,579 / 1,412

Values are HRs (95% CIs) unless otherwise indicated. Cox models used age as the underlying time scale with delayed entry

*HR* Hazard ratio, *CI* Confidence interval

**Fig. 2:**
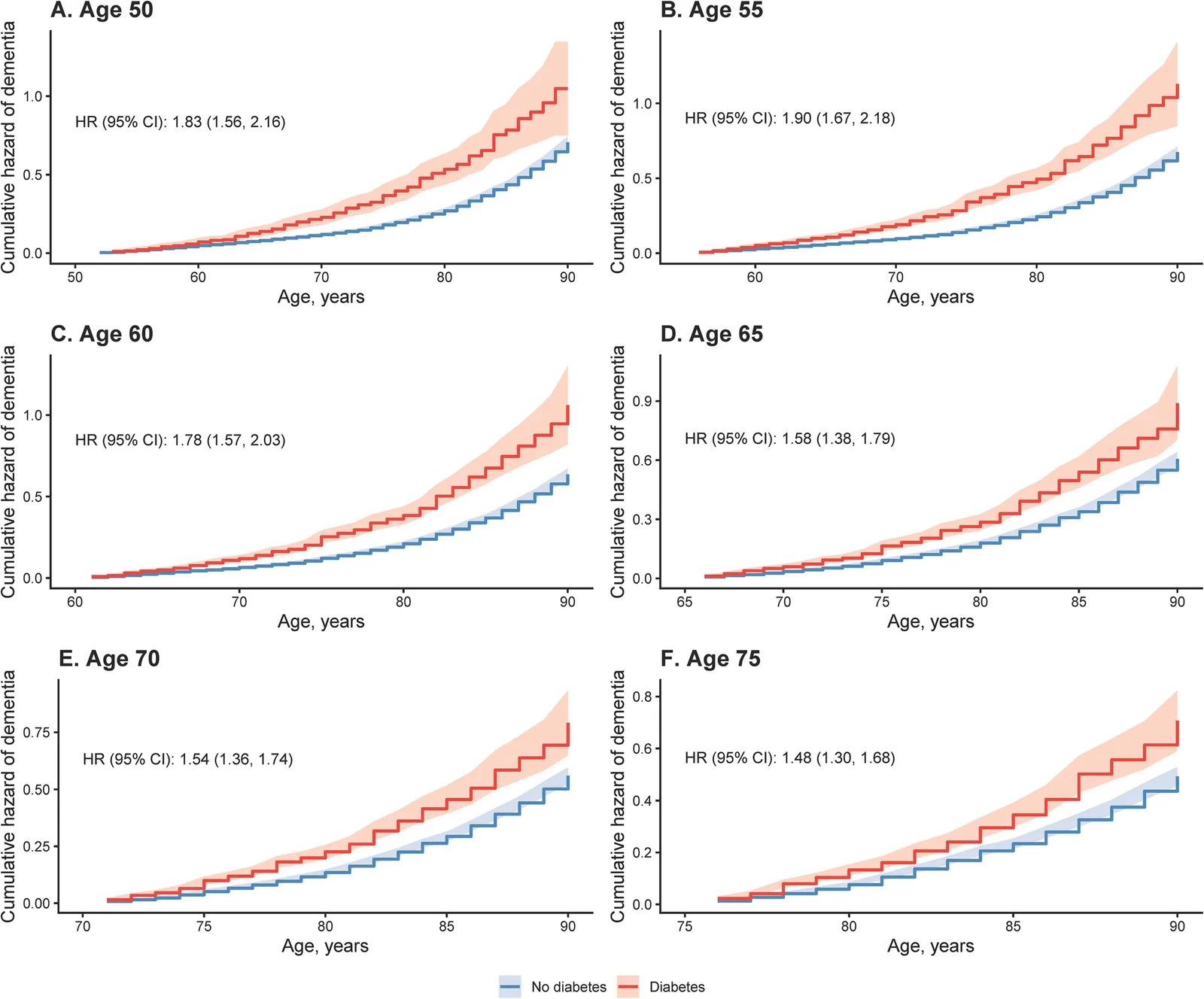
Cumulative hazard of incident dementia by diabetes status at prespecified landmark ages. Panels **A**–**F **show cumulative hazards at landmark ages 50, 55, 60, 65, 70 and 75 years. Curves and displayed HRs are unadjusted. Cox models used age as the underlying time scale with delayed entry

Diabetes associations attenuated with age in both the pooled landmark model (ratio of HRs per 5-year increase in landmark age, 0.95; 95% CI, 0.92–0.99; *P* = .013) and the time-updated model (ratio of HRs per 5-year increase in attained age, 0.93; 95% CI, 0.89–0.97; *P* < .001). The proportional hazards assumption was not met for time-updated diabetes; its primary HR was therefore interpreted as a follow-up-averaged association. Exposure-specific proportionality was supported at all landmarks except age 70 years (eTable 3). Cumulative hazard curves showed consistently higher dementia hazards among participants with diabetes (Fig. 2).

### Estimated time since first diabetes ascertainment, competing risk, mediation and sensitivity analyses

An estimated interval exceeding 10 years since first diabetes ascertainment was associated with higher dementia risk at all landmark ages (eTable 4). In fully adjusted models, HRs for this category ranged from 1.28 to 1.64. Associations for shorter intervals were less consistent, and category-specific estimates were not uniformly monotonic.

When death was treated as a competing event, diabetes remained associated with higher cumulative incidence of incident dementia at all landmark ages (eTable 5). Subdistribution hazard ratios were attenuated compared with Cox estimates but remained statistically significant, suggesting that competing mortality influenced, but did not eliminate, the association.

Exploratory mediation analyses identified modest indirect associations through the composite indicator of heart disease or stroke (eTable 6). The estimated mediated proportion was 15.9% at age 50 years, 13.6% at age 55 years, 13.6% at age 60 years, 14.3% at age 65 years, 12.6% at age 70 years and 8.8% at age 75 years. These estimates were not interpreted as evidence of causal mediation.

Sensitivity analyses excluding participants who developed dementia within 2 years after risk-set entry produced broadly consistent associations (eTable 7). Excluding 429 participants identified solely through elevated HbA1c produced a similar association (HR, 1.31; 95% CI, 1.20–1.43; eTable 8).

The baseline HbA1c subset included 6,872 participants and was broadly comparable with the full cohort. Median age was 64 years in both groups, and diabetes prevalence was 35.8% in both groups. Incident dementia occurred in 11.8% of the HbA1c subset and 12.5% of the full cohort (eTable 9). Among participants with baseline HbA1c data, each 1%-point higher HbA1c was associated with an 8% higher dementia risk (HR, 1.08; 95% CI, 1.01–1.15; *P* = .021). HbA1c of 6.5% or higher was also associated with higher incident dementia risk (HR, 1.34; 95% CI, 1.12–1.60; *P* = .001; eTable 10).

## Discussion

### Principal findings

In this nationally representative prospective cohort, diabetes was associated with higher incident dementia risk classified by the Langa–Weir algorithm. The association was observed when diabetes was modelled as a time-updated exposure and across landmark ages from 50 to 75 years. Although the association attenuated at older landmark ages, it remained statistically significant at age 75 years. An estimated interval exceeding 10 years since first diabetes ascertainment was associated with higher risk at each landmark. These findings are observational associations and should not be interpreted as evidence that diabetes causes dementia.

These findings suggest that the strength of the diabetes–dementia association varies across later life. This variation may reflect attained age, cumulative metabolic exposure, competing mortality and selective survival. Fine-Gray models showed that the association persisted after accounting for death as a competing event, whereas exploratory mediation analyses identified only modest indirect associations through heart disease or stroke.

### Comparison with previous studies

Our findings are consistent with previous evidence linking diabetes to cognitive decline, all-cause dementia, Alzheimer’s disease, and vascular dementia [6, 7, 17, 18]. They also align with studies showing higher dementia risk among people with earlier diabetes onset or longer diabetes duration [12, 14]. The present analysis extends this work by combining time-updated exposure modelling, conditional landmark cohorts and formal age-interaction tests in a large general population sample. Both interaction analyses indicated that the relative association attenuated with increasing attained age.

The time-updated Cox and landmark analyses addressed distinct but complementary questions. The time-updated model related dementia risk to current diabetes status and allowed exposure to change after first ascertainment. In each age-anchored landmark analysis, diabetes status and the estimated interval since first ascertainment were defined at the prespecified landmark age and held fixed. Risk-set entry occurred at the later of that age or the participant’s wave 10 age. Participants were required to be alive, dementia-free and under observation at entry. These delayed-entry landmark models therefore provided conditional estimates beyond the overall time-updated association. However, older risk sets selected survivors who remained observable. Participants who died, developed dementia or left follow-up before entry could not contribute. Changes in cohort composition may therefore partly explain attenuation at older landmarks. Because the proportional hazards assumption was not met for time-updated diabetes, its primary HR represents a follow-up-averaged association. The diabetes-by-age interaction provides more direct evidence of age-related variation. Landmark HRs should likewise be interpreted as average conditional associations within their respective risk sets.

### Potential mechanisms

The exploratory mediation findings were compatible with a possible contribution from vascular pathways, but they did not establish a causal mechanism. Chronic hyperglycaemia, insulin resistance, dyslipidaemia, and advanced glycation end-products promote endothelial dysfunction, oxidative stress, impaired nitric oxide bioavailability, and disrupted cerebral blood-flow regulation [9, 19–27]. Diabetes-related inflammation and matrix remodelling may also impair tight junctions and blood–brain barrier integrity [28, 29]. Entry of plasma proteins and inflammatory mediators into brain tissue can amplify neuroinflammation and neurovascular dysfunction [30–33]. These processes may promote cerebral small-vessel disease, microinfarcts, white-matter injury, and hypoperfusion [34–36].

The statistically detectable but modest indirect associations suggest that heart disease or stroke accounted for only a limited proportion of the overall association. These estimates depend on strong assumptions concerning temporal ordering, correct model specification and the absence of unmeasured confounding. They should therefore be interpreted as exploratory rather than as evidence that these conditions causally mediate the diabetes–dementia relationship.

Non-vascular mechanisms may also contribute [37, 38]. Brain insulin resistance can disrupt IRS-1/PI3K/Akt signalling, synaptic plasticity, neuronal survival, and energy metabolism [38–40]. It may also impair amyloid-β clearance and promote tau phosphorylation [41, 42]. Hyperglycaemia and elevated fatty acids can increase mitochondrial oxidative stress and lipid peroxidation, generating toxic aldehydes that damage membranes, organelles, and synapses [43–45]. Excess hexosamine-pathway activity and altered O-GlcNAcylation may further disturb calcium homeostasis and neuronal transcriptional networks [46–49].

The observed associations are consistent with several vascular and non-vascular hypotheses, but the present data cannot establish the underlying biological pathways. These mechanisms require confirmation in studies with imaging, biomarker, cerebrovascular, and dementia-subtype data.

### Competing mortality and age-dependent associations

The attenuation of associations at older ages may partly reflect competing mortality and selective survival. Diabetes increases the risk of mortality, cardiovascular events, and multimorbidity [15, 16, 50]. Some older adults with diabetes may die or develop severe comorbidity before dementia is observed, which can attenuate estimated dementia associations in late life [50, 51].

Selective entry into the landmark cohorts may also contribute. Each successively older cohort excluded participants who had died, developed dementia or left observation before risk-set entry. Consequently, participants with diabetes who survived to older landmark ages may have represented a healthier or more intensively treated subgroup.

Fine–Gray analyses showed that diabetes remained associated with higher cumulative dementia incidence after death was modelled as a competing event. Therefore, attenuation at older ages should not be interpreted as disappearance of diabetes-related cognitive risk. A more cautious interpretation is that late-life dementia risk among individuals with diabetes reflects cumulative metabolic exposure, cardiovascular complications, multimorbidity, competing death and selective survival [12, 51, 52]. Competing-risk modelling does not, however, eliminate selection arising from survival to an older landmark age.

### Public health and clinical implications

The results support a life-course approach to cognitive risk assessment in people with diabetes. Current diabetes status alone may miss important information carried by the assigned exposure-transition age, estimated time since first diabetes ascertainment, cardiovascular or cerebrovascular disease and competing health risks. People with earlier diabetes ascertainment or longer estimated interval may warrant closer cognitive monitoring and more integrated cardiometabolic risk management. However, the assigned transition age is not a clinically verified onset age, and the estimated interval should not be interpreted as true disease duration.

At the same time, dementia prevention in older adults with diabetes should be individualised. Glycaemic targets and vascular risk management should account for life expectancy, functional status, hypoglycaemia risk, cardiovascular burden, cognitive status and patient preferences, rather than applying one target without considering overall health [53, 54]. The present observational findings do not demonstrate that intensive glycaemic treatment or cardiovascular risk management prevents dementia.

### Strengths and limitations

This study has several strengths, including a nationally representative longitudinal cohort, large sample size, long follow-up, repeated diabetes ascertainment and complementary time-updated and landmark analyses. Competing-risk, age-interaction and sensitivity analyses further assessed the robustness and age dependence of the associations. 

The study also has limitations. Dementia was classified algorithmically rather than by clinical diagnosis or standardised neuropsychological assessment, so outcome misclassification is possible. The Langa–Weir algorithm cannot distinguish Alzheimer’s disease from vascular or other dementias, limiting interpretation of proposed vascular pathways. The dataset did not provide a verified clinical diabetes-onset date. The assigned exposure-transition age was based on the first interview or biomarker assessment meeting the diabetes criteria. Biennial assessments and missed waves may therefore have delayed ascertainment of both diabetes and dementia. The estimated interval represents time since first ascertainment rather than true disease duration. HbA1c was available only during waves 10 to 13 and in a subset of participants. Missing HbA1c was not interpreted as absence of diabetes, and previously identified diabetes status was carried forward. The estimated interval and transition age were mathematically linked within each landmark and could not be estimated independently. Differential dementia ascertainment also cannot be excluded. Scheduled HRS assessments may reduce surveillance bias associated with healthcare contact, but diabetes may affect interview participation, proxy use, test completion and attrition. More complete ascertainment among participants with diabetes could inflate the association, whereas differential loss to follow-up could attenuate it. Selective survival may also have contributed to attenuation in older risk sets. Because proportional hazards were not supported for time-updated diabetes, its primary HR should be interpreted as a follow-up-averaged association. Residual confounding by healthcare access, socioeconomic conditions, diet, physical activity, sleep, depressive symptoms, kidney function, APOE genotype, long-term glycaemic control and treatment cannot be excluded. The observational design cannot establish causality, and the mediation assumptions cannot be fully verified. These analyses should therefore be considered exploratory rather than evidence of causal pathways. Finally, the absence of neuroimaging, cerebrovascular markers and Alzheimer’s disease-related biomarkers limited mechanistic inference.

## Conclusions

Diabetes was associated with higher incident dementia risk in the HRS cohort across landmark ages from 50 to 75 years. An estimated interval exceeding 10 years since first diabetes ascertainment was associated with higher risk at each landmark. The association remained evident after accounting for death as a competing event, although attenuation at older ages may partly reflect competing mortality, selective survival and changing cohort composition.

Exploratory mediation analyses identified modest indirect associations through heart disease or stroke but did not establish causal mediation. Cognitive risk assessment in people with diabetes may consider the timing of ascertainment, estimated time since ascertainment, vascular burden and competing mortality. These observational findings should inform risk assessment and future research, rather than be interpreted as evidence that diabetes or its vascular complications directly cause dementia.

## Supplementary Information

Supplementary Material 1.

## Data Availability

The Health and Retirement Study data are publicly available from the HRS website (https://hrs.isr.umich.edu/data-products) subject to HRS data-use requirements.
