# Supplementary material for: Timing and duration of diabetes and incident dementia in older adults: a prospective cohort study
Source: Alzheimers Res Ther. 2026 Oct 10;18:234. doi: 10.1186/s13195-026-02189-z (PMC13673855; doi:10.1186/s13195-026-02189-z)
Supplement: Supplementary file 1 [file 13195_2026_2189_MOESM1_ESM.docx]

**eTable 1. Wave-Specific Participation and Data Availability in the Final Analytic Cohorts**

| HRS wave | Year | Final analytic cohort interviewed, No. | Cognitive classification available, No. | Diabetes interview/medication data available, No. | HbA1c available, No. |
| --- | --- | --- | --- | --- | --- |
| 10 | 2010 | 18,028 | 18,028 | 18,028 | 6,872 |
| 11 | 2012 | 17,623 | 17,622 | 17,623 | 6,408 |
| 12 | 2014 | 16,082 | 16,082 | 16,082 | 6,410 |
| 13 | 2016 | 14,210 | 14,210 | 14,210 | 4,524 |
| 14 | 2018 | 11,678 | 11,678 | 11,678 | Not collected |
| 15 | 2020 | 10,687 | 10,687 | 10,687 | Not collected |

Note: Final analytic-cohort counts refer to the 18028 participants included in the primary analysis. These columns describe participation and should not be used to reconstruct analysis-specific exclusions in Figure 1 or the separate time-updated sensitivity analysis. Diabetes status was ascertainable when self-reported physician diagnosis, glucose-lowering medication information or HbA1c, when available, permitted classification. Missing HbA1c alone was not interpreted as absence of diabetes. Cognitive classifications were obtained from the HRS Langa–Weir final imputed classification data. HbA1c was available only in subsets during 2010–2016 and was not collected in 2018 or 2020.

**eTable 2. Characteristics of the Prespecified Landmark Cohorts**

| Characteristic | Landmark 50 y | Landmark 55 y | Landmark 60 y | Landmark 65 y | Landmark 70 y | Landmark 75 y |
| --- | --- | --- | --- | --- | --- | --- |
| Participants, n | 18,028 | 17,820 | 16,650 | 13,114 | 10,033 | 7,579 |
| Age at risk-set entry, median (IQR), y | 64.0 (56.0-73.0) | 64.0 (56.0-73.0) | 66.0 (60.0-74.0) | 70.0 (65.0-76.0) | 72.0 (70.0-78.0) | 75.0 (75.0-80.0) |
| Women, No. (%) | 10,351 (57.4%) | 10,243 (57.5%) | 9,580 (57.5%) | 7,573 (57.7%) | 5,811 (57.9%) | 4,397 (58.0%) |
| University degree, No. (%) | 8,811 (48.9%) | 8,709 (48.9%) | 8,159 (49.0%) | 6,203 (47.3%) | 4,516 (45.0%) | 3,274 (43.2%) |
| Black/African American, No. (%) | 3,354 (18.6%) | 3,296 (18.5%) | 2,938 (17.6%) | 2,033 (15.5%) | 1,308 (13.0%) | 887 (11.7%) |
| Married, No. (%) | 7,280 (40.4%) | 7,196 (40.4%) | 6,695 (40.2%) | 5,279 (40.3%) | 4,068 (40.5%) | 3,257 (43.0%) |
| Current smoker, No. (%) | 2,762 (15.3%) | 2,699 (15.1%) | 2,363 (14.2%) | 1,501 (11.4%) | 920 (9.2%) | 526 (6.9%) |
| Alcohol consumption, No. (%) | 10,501 (58.2%) | 10,360 (58.1%) | 9,589 (57.6%) | 7,186 (54.8%) | 5,226 (52.1%) | 3,837 (50.6%) |
| No diabetes, No. (%) | 16,837 (93.4%) | 15,722 (88.2%) | 13,862 (83.3%) | 10,595 (80.8%) | 7,826 (78.0%) | 5,679 (74.9%) |
| Estimated time since first diabetes ascertainment <=5 y, No. (%) | 583 (3.2%) | 1,059 (5.9%) | 1,142 (6.9%) | 919 (7.0%) | 834 (8.3%) | 666 (8.8%) |
| Estimated time since first diabetes ascertainment >5-10 y, No. (%) | 253 (1.4%) | 440 (2.5%) | 728 (4.4%) | 594 (4.5%) | 457 (4.6%) | 454 (6.0%) |
| Estimated time since first diabetes ascertainment >10 y, No. (%) | 355 (2.0%) | 599 (3.4%) | 918 (5.5%) | 1,006 (7.7%) | 916 (9.1%) | 780 (10.3%) |
| Chronic lung disease, No. (%) | 2,938 (16.3%) | 2,923 (16.4%) | 2,766 (16.6%) | 2,321 (17.7%) | 1,861 (18.5%) | 1,428 (18.8%) |
| Cancer, No. (%) | 3,777 (21.0%) | 3,751 (21.0%) | 3,632 (21.8%) | 3,191 (24.3%) | 2,691 (26.8%) | 2,157 (28.5%) |
| Dementia events, No. (%) | 2,247 (12.5%) | 2,209 (12.4%) | 2,044 (12.3%) | 1,852 (14.1%) | 1,673 (16.7%) | 1,412 (18.6%) |
| Follow-up, mean (SD), y | 8.04 (2.70) | 7.65 (2.73) | 6.74 (3.07) | 6.42 (3.12) | 6.27 (3.09) | 5.63 (2.90) |

Note: Each age-anchored landmark cohort included participants who were alive, dementia-free and under observation at risk-set entry. Risk-set entry occurred at the later of the prespecified landmark age or wave 10 age. Diabetes status and estimated time since first ascertainment were defined at the prespecified landmark age and held fixed during post-entry follow-up. Values are No. (%) unless otherwise indicated. IQR indicates interquartile range; SD, standard deviation.

**eTable 3. Proportional Hazards Diagnostics and Age-Interaction Analyses**

| Model | Diabetes PH-test P | Global PH-test P | Age interaction | Interaction P |
| --- | --- | --- | --- | --- |
| Time-updated Cox | <.001 | <.001 | Ratio of HRs per 5 y, 0.93 (0.89-0.97) | <.001 |
| Landmark age 50 y | 0.430 | <.001 | Not applicable |  |
| Landmark age 55 y | 0.269 | <.001 | Not applicable |  |
| Landmark age 60 y | 0.212 | <.001 | Not applicable |  |
| Landmark age 65 y | 0.197 | <.001 | Not applicable |  |
| Landmark age 70 y | 0.038 | <.001 | Not applicable |  |
| Landmark age 75 y | 0.331 | 0.027 | Not applicable |  |
| Pooled landmark interaction | Not applicable | Not applicable | Ratio of HRs per 5-y older landmark, 0.95 (0.92-0.99) | 0.013 |

Note: Proportional hazards were assessed using tests based on scaled Schoenfeld residuals. In the time-updated Cox model, the diabetes-specific and global tests indicated non-proportionality; therefore, the constant diabetes HR represents a follow-up-averaged association, and the diabetes-by-attained-age interaction is emphasised. The interaction estimate of 0.93 indicates that the diabetes-associated HR was multiplied by 0.93 for each 5-year increase in attained age. A diabetes-by-landmark-age interaction could not be estimated within an individual landmark model because landmark age was fixed by design. It was therefore evaluated in the pooled landmark analysis; the estimate of 0.95 represents the multiplicative change in the diabetes-associated HR per 5-year older landmark age. PH indicates proportional hazards; HR, hazard ratio.

**eTable 4. Association Between Estimated Time Since First Diabetes Ascertainment and Incident Dementia at Prespecified Landmark Ages**

| Landmark age,y | Estimated interval since first ascertainment | Model 1, HR (95% CI) | P value | Model 2, HR (95% CI) | P value | Model 3, HR (95% CI) | P value |
| --- | --- | --- | --- | --- | --- | --- | --- |
| 50 | No diabetes | 1 [Reference] |  | 1 [Reference] |  | 1 [Reference] |  |
|  | ≤5 years | 1.59 (1.26–2.01) | <.001 | 1.54 (1.22–1.94) | <.001 | 1.54 (1.22–1.94) | <.001 |
|  | >5–10 years | 1.27 (0.88–1.83) | 0.211 | 1.25 (0.86–1.80) | 0.241 | 1.24 (0.86–1.79) | 0.259 |
|  | >10 years | 1.58 (1.21–2.07) | <.001 | 1.59 (1.21–2.08) | <.001 | 1.58 (1.21–2.07) | <.001 |
|  | P for trend |  | <.001 |  | <.001 |  | <.001 |
| 55 | No diabetes | 1 [Reference] |  | 1 [Reference] |  | 1 [Reference] |  |
|  | ≤5 years | 1.57 (1.31–1.89) | <.001 | 1.53 (1.27–1.83) | <.001 | 1.52 (1.27–1.83) | <.001 |
|  | >5–10 years | 1.62 (1.24–2.13) | <.001 | 1.55 (1.18–2.03) | 0.002 | 1.55 (1.18–2.04) | 0.002 |
|  | >10 years | 1.54 (1.23–1.93) | <.001 | 1.54 (1.23–1.92) | <.001 | 1.53 (1.22–1.91) | <.001 |
|  | P for trend |  | <.001 |  | <.001 |  | <.001 |
| 60 | No diabetes | 1 [Reference] |  | 1 [Reference] |  | 1 [Reference] |  |
|  | ≤5 years | 1.31 (1.07–1.60) | 0.008 | 1.26 (1.04–1.54) | 0.021 | 1.26 (1.03–1.54) | 0.022 |
|  | >5–10 years | 1.59 (1.27–2.00) | <.001 | 1.54 (1.22–1.93) | <.001 | 1.53 (1.22–1.93) | <.001 |
|  | >10 years | 1.68 (1.39–2.04) | <.001 | 1.64 (1.35–1.99) | <.001 | 1.64 (1.35–1.98) | <.001 |
|  | P for trend |  | <.001 |  | <.001 |  | <.001 |
| 65 | No diabetes | 1 [Reference] |  | 1 [Reference] |  | 1 [Reference] |  |
|  | ≤5 years | 0.99 (0.79–1.25) | 0.951 | 0.97 (0.77–1.23) | 0.811 | 0.97 (0.77–1.23) | 0.802 |
|  | >5–10 years | 1.37 (1.08–1.74) | 0.01 | 1.32 (1.04–1.67) | 0.025 | 1.32 (1.03–1.67) | 0.025 |
|  | >10 years | 1.65 (1.39–1.96) | <.001 | 1.59 (1.33–1.89) | <.001 | 1.59 (1.33–1.89) | <.001 |
|  | P for trend |  | <.001 |  | <.001 |  | <.001 |
| 70 | No diabetes | 1 [Reference] |  | 1 [Reference] |  | 1 [Reference] |  |
|  | ≤5 years | 1.28 (1.06–1.54) | 0.011 | 1.22 (1.01–1.48) | 0.036 | 1.22 (1.01–1.48) | 0.036 |
|  | >5–10 years | 1.07 (0.80–1.43) | 0.664 | 1.04 (0.77–1.39) | 0.806 | 1.04 (0.77–1.39) | 0.811 |
|  | >10 years | 1.56 (1.32–1.84) | <.001 | 1.48 (1.26–1.75) | <.001 | 1.48 (1.26–1.75) | <.001 |
|  | P for trend |  | <.001 |  | <.001 |  | <.001 |
| 75 | No diabetes | 1 [Reference] |  | 1 [Reference] |  | 1 [Reference] |  |
|  | ≤5 years | 1.35 (1.12–1.63) | 0.002 | 1.32 (1.10–1.60) | 0.003 | 1.32 (1.10–1.60) | 0.003 |
|  | >5–10 years | 1.25 (0.97–1.60) | 0.088 | 1.20 (0.93–1.54) | 0.16 | 1.20 (0.93–1.55) | 0.159 |
|  | >10 years | 1.35 (1.12–1.61) | 0.001 | 1.28 (1.07–1.54) | 0.007 | 1.28 (1.07–1.54) | 0.007 |
|  | P for trend |  | <.001 |  | <.001 |  | <.001 |

Note:Values are HRs (95% CIs) unless otherwise indicated. Cox proportional hazards models used age as the underlying time scale with delayed entry. At each landmark, estimated time since first diabetes ascertainment was calculated as the prespecified landmark age minus the assigned exposure-transition age. It was categorised as 5 years or less, more than 5 to 10 years, or more than 10 years. Participants without diabetes ascertainment by the corresponding landmark age served as the reference group. Incident dementia was defined using the Langa–Weir classification algorithm. Model 1 adjusted for education, race or ethnicity, marital status and sex. Model 2 additionally adjusted for alcohol use and smoking status. Model 3 additionally adjusted for cancer and chronic lung disease. P for trend was calculated by modelling the categories as an ordinal variable. Death was treated as a censoring event. CI indicates confidence interval; HR, hazard ratio.

**eTable 5. Association Between Diabetes and Incident Dementia Using Fine-Gray Competing Risk Models at Prespecified Landmark Ages**

| Landmark age,y | Follow-up, mean (SD), y | Model 1, SHR (95% CI) | P value | Model 2, SHR (95% CI) | P value | Model 3, SHR (95% CI) | P value |
| --- | --- | --- | --- | --- | --- | --- | --- |
| 50 | 8.04 (2.70) | 1.43 (1.21–1.68) | <.001 | 1.40 (1.19–1.65) | <.001 | 1.40 (1.19–1.65) | <.001 |
| 55 | 7.65 (2.73) | 1.50 (1.31–1.71) | <.001 | 1.46 (1.28–1.67) | <.001 | 1.46 (1.28–1.67) | <.001 |
| 60 | 6.74 (3.07) | 1.45 (1.27–1.64) | <.001 | 1.41 (1.24–1.60) | <.001 | 1.41 (1.24–1.60) | <.001 |
| 65 | 6.42 (3.12) | 1.28 (1.12–1.45) | <.001 | 1.24 (1.09–1.41) | <.001 | 1.24 (1.09–1.41) | <.001 |
| 70 | 6.27 (3.09) | 1.30 (1.15–1.46) | <.001 | 1.25 (1.10–1.41) | <.001 | 1.25 (1.10–1.41) | <.001 |
| 75 | 5.63 (2.90) | 1.25 (1.11–1.42) | <.001 | 1.21 (1.07–1.38) | <.001 | 1.21 (1.07–1.38) | <.001 |

Note: Values are SHRs (95% CIs) unless otherwise indicated. Fine–Gray subdistribution hazard models used follow-up time from risk-set entry, with death treated as a competing event. Models adjusted for age at risk-set entry. At each landmark, diabetes status was defined by whether diabetes had first been ascertained on or before that age. Participants without diabetes ascertainment by the corresponding landmark age served as the reference group. Incident dementia was defined using the Langa–Weir classification algorithm. Model 1 adjusted for education, race or ethnicity, marital status and sex. Model 2 additionally adjusted for alcohol use and smoking status. Model 3 additionally adjusted for cancer and chronic lung disease.

Abbreviations: CI, confidence interval; SHR, subdistribution hazard ratio.

**eTable 6. Exploratory Mediation of the Association Between Diabetes and Incident Dementia Through a Composite Indicator of Heart Disease or Stroke**

| Landmark age,y | 50 | 55 | 60 | 65 | 70 | 75 |
| --- | --- | --- | --- | --- | --- | --- |
| No. of participants | 18,028 | 17,820 | 16,650 | 13,114 | 10,033 | 7,579 |
| Total effect, HR (95% CI) | 1.53 (1.30-1.81); P <.001 | 1.56 (1.37-1.79); P <.001 | 1.47 (1.29-1.67); P <.001 | 1.32 (1.15-1.50); P <.001 | 1.29 (1.14-1.47); P <.001 | 1.26 (1.11-1.44); P <.001 |
| Controlled direct effect | 1.45 (1.23-1.71); P <.001 | 1.49 (1.30-1.70); P <.001 | 1.41 (1.24-1.60); P <.001 | 1.27 (1.11-1.45); P <.001 | 1.26 (1.11-1.43); P <.001 | 1.24 (1.09-1.41); P= .001 |
| Natural indirect effect, HR (95% CI) | 1.06 (1.04-1.08); P <.001 | 1.05 (1.03-1.07); P <.001 | 1.05 (1.03-1.06); P <.001 | 1.04 (1.02-1.05); P <.001 | 1.03 (1.02-1.04); P <.001 | 1.02 (1.01-1.03); P <.001 |
| Proportion mediated, % (95% CI) | 15.9% (9.0-22.8) | 13.6% (8.3-19.0) | 13.6% (7.7-19.4) | 14.3% (6.3-22.4) | 12.6% (4.9-20.2) | 8.8% (2.3-15.2) |

Note: Values are HRs (95% CIs) unless otherwise indicated. Counterfactual mediation analyses evaluated a composite indicator of heart disease or stroke and allowed an exposure–mediator interaction. Models adjusted for education, race or ethnicity, marital status, sex, alcohol use, smoking status, cancer and chronic lung disease. Interpretation requires consistency and positivity, correct model specification and temporal ordering, no unmeasured exposure–outcome, exposure–mediator or mediator–outcome confounding, and no mediator–outcome confounder affected by diabetes. These assumptions cannot be fully verified in the observational HRS data. The estimates are exploratory and should not be interpreted as definitive causal evidence. CI indicates confidence interval; HR, hazard ratio.

Abbreviations: CI, confidence interval; HR, hazard ratio.

**eTable 7. Association of Diabetes With Incident Dementia After Excluding Participants With Dementia Within 2 Years After Risk-Set Entry**

| Landmark age,y | Follow-up, mean (SD), y | Model 1, HR (95% CI) | P value | Model 2, HR (95% CI) | P value | Model 3, HR  (95% CI) | P value |
| --- | --- | --- | --- | --- | --- | --- | --- |
| 50 | 8.27 (2.48) | 1.49 (1.22–1.83) | <.001 | 1.47 (1.20–1.80) | <.001 | 1.46 (1.20–1.79) | <.001 |
| 55 | 7.85 (2.55) | 1.63 (1.39–1.91) | <.001 | 1.60 (1.36–1.87) | <.001 | 1.59 (1.36–1.86) | <.001 |
| 60 | 6.90 (2.99) | 1.59 (1.37–1.84) | <.001 | 1.55 (1.33–1.80) | <.001 | 1.54 (1.33–1.79) | <.001 |
| 65 | 6.60 (3.05) | 1.46 (1.26–1.70) | <.001 | 1.43 (1.23–1.66) | <.001 | 1.43 (1.23–1.66) | <.001 |
| 70 | 6.48 (3.01) | 1.48 (1.28–1.72) | <.001 | 1.44 (1.24–1.67) | <.001 | 1.44 (1.24–1.67) | <.001 |
| 75 | 5.83 (2.84) | 1.39 (1.20–1.62) | <.001 | 1.36 (1.17–1.58) | <.001 | 1.36 (1.17–1.58) | <.001 |

Note: Values are HRs (95% CIs) unless otherwise indicated. Participants who developed dementia within 2 years after risk-set entry were excluded. Cox proportional hazards models used age as the underlying time scale with delayed entry. At each landmark, diabetes status was defined by whether diabetes had first been ascertained on or before that age. Participants without diabetes ascertainment by the corresponding landmark age served as the reference group. Incident dementia was defined using the Langa–Weir classification algorithm. Model 1 adjusted for education, race or ethnicity, marital status and sex. Model 2 additionally adjusted for alcohol use and smoking status. Model 3 additionally adjusted for cancer and chronic lung disease. Death was treated as a censoring event.

Abbreviations: CI, confidence interval; HR, hazard ratio.

**eTable 8. Sensitivity Analyses of Time-Updated Diabetes and Incident Dementia**

| **Analysis** | **Participants, No.** | **Dementia events, No.** | **HR (95% CI)** | **P value** |
| --- | --- | --- | --- | --- |
| Primary | 18,028 | 2,247 | 1.29 (1.19-1.41) | <.001 |
| Exclude HbA1c-only diabetes ascertainment | 17,599 | 2,209 | 1.31 (1.20-1.43) | <.001 |

Note: Fully adjusted Cox models used attained age as the time scale and adjusted for education, race or ethnicity, marital status, sex, alcohol consumption, smoking, cancer, and chronic lung disease. HR indicates hazard ratio; CI, confidence interval.

**eTable 9. Characteristics of the Baseline HbA1c Subset and Full Analytic Cohort**

| Characteristic | Full analytic cohort | Baseline HbA1c subset |
| --- | --- | --- |
| Participants, n | 18,028 | 6,872 |
| Age, median (IQR), y | 64.0 (56.0-73.0) | 64.0 (56.0-73.0) |
| Women, No. (%) | 10,351 (57.4%) | 4,002 (58.2%) |
| University degree, No. (%) | 8,811 (48.9%) | 3,383 (49.2%) |
| Black/African American, No. (%) | 3,354 (18.6%) | 1,218 (17.7%) |
| Married, No. (%) | 7,280 (40.4%) | 2,756 (40.1%) |
| Current smoker, No. (%) | 2,762 (15.3%) | 995 (14.5%) |
| Alcohol consumption, No. (%) | 10,501 (58.2%) | 4,038 (58.8%) |
| Diabetes, No. (%) | 6,446 (35.8%) | 2,461 (35.8%) |
| Hypertension, No. (%) | 14,097 (78.2%) | 5,482 (79.8%) |
| Heart disease, No. (%) | 6,467 (35.9%) | 2,524 (36.7%) |
| Stroke, No. (%) | 2,234 (12.4%) | 830 (12.1%) |
| Chronic lung disease, No. (%) | 2,938 (16.3%) | 1,140 (16.6%) |
| Cancer, No. (%) | 3,777 (21.0%) | 1,424 (20.7%) |
| Incident dementia, No. (%) | 2,247 (12.5%) | 812 (11.8%) |
| Follow-up, median (IQR), y | 10.0 (6.0-10.0) | 10.0 (6.0-10.0) |

Note: Values are No. (%) unless otherwise indicated. The HbA1c subset was nested within the full analytic cohort and is described to assess potential selection into biomarker availability.

**eTable 10. Association Between HbA1c and Incident Dementia**

| HbA1c Exposure | No. of Participants | Model 1 HR (95% CI) | P Value | Model 2 HR (95% CI) | P Value | Model 3 HR (95% CI) | P Value |
| --- | --- | --- | --- | --- | --- | --- | --- |
| HbA1c, per 1% increase | 6872 | 1.09 (1.03-1.16) | 0.005 | 1.08 (1.01-1.15) | 0.02 | 1.08 (1.01-1.15) | 0.021 |
| HbA1c Category |  |  |  |  |  |  |  |
| HbA1c <6.5% | 5921 | 1 [Reference] |  | 1 [Reference] |  | 1 [Reference] |  |
| HbA1c ≥6.5% | 951 | 1.39 (1.17-1.66) | <.001 | 1.35 (1.13-1.61) | 0.001 | 1.34 (1.12-1.60) | 0.001 |

Note: HR indicates hazard ratio; CI, confidence interval; HbA1c, haemoglobin A1c. Cox proportional hazards models used age as the time scale, with entry and exit ages defining the start and stop times. HbA1c was categorised as <6.5% and ≥6.5%, with HbA1c <6.5% as the reference group. Model 1 adjusted for sex, education, race or ethnicity and marital status. Model 2 additionally adjusted for alcohol consumption and smoking status. Model 3 further adjusted for chronic lung disease and cancer.

Figure S1. Cumulative incidence of incident dementia by diabetes status at prespecified landmark ages, accounting for death as a competing event. Panels A–F show cumulative incidence at landmark ages 50, 55, 60, 65, 70 and 75 years. Red lines indicate participants whose diabetes was first ascertained on or before the corresponding landmark age, and blue lines indicate those without diabetes ascertainment by that age. Shaded areas represent 95% CIs. Displayed SHRs and 95% CIs were estimated using unadjusted Fine–Gray models with follow-up time since risk-set entry as the time scale.
